# Global Status of Adult Immunization Post COVID-19 Pandemic

**DOI:** 10.3390/vaccines13040401

**Published:** 2025-04-11

**Authors:** Alba Vilajeliu, Victor Vega, Randie Gibson, Francisco Nogareda, Xiaojun Wang, Donald J. Brooks, Charles Shey Wiysonge, Osman Niyazi Cakmak, Osama Mere, Melanie Marti, Phillip Lambach, Stephanie Shendale, Marcela Contreras, Emmanuel Njambe, Erin Grace Sparrow, Joachim Hombach, Ann Lindstrand

**Affiliations:** 1 Department of Immunization, Vaccines and Biologicals (IVB), World Health Organization, 1211 Geneva, Switzerland; vegav@who.int (V.V.); gibsonr@who.int (R.G.); martim@who.int (M.M.); lambachp@who.int (P.L.); shendales@who.int (S.S.); sparrowe@who.int (E.G.S.); hombachj@who.int (J.H.); lindstranda@who.int (A.L.); 2 Special Program Comprehensive Immunization, Pan American Health Organization (PAHO)/World Health Organization Regional Office for the Americas, Washington, DC 20037, USA; nogaredfra@paho.org (F.N.); contrermar@paho.org (M.C.); 3 Vaccine-Preventable Diseases and Immunization Unit, Division of Programmes for Disease Control, World Health Organization, Regional Office for Western Pacific, Manila 1000, Philippines; wangxia@who.int; 4 Department of Epidemic and Pandemic Threat Management, World Health Organization, 1211 Geneva, Switzerland; brooksd@who.int; 5 Vaccine-Preventable Diseases Programme, World Health Organization, Regional Office for Africa, Brazzaville P.O. Box 06, Congo; sheyc@who.int; 6 Vaccine-Preventable Diseases and Immunization Programme, World Health Organization, Regional Office for Europe, 2100 Copenhagen, Denmark; cakmako@who.int; 7 Immunization Vaccine Preventable Disease and Polio Transition Unit, Department of Communicable Diseases and Universal Health Coverage, World Health Organization, Regional Office for the Eastern Mediterranean, Cairo 11371, Egypt; mereo@who.int; 8 Immunizations and Vaccines Development, World Health Organization, Regional Office for South-East Asia, New Delhi 110002, India; emmanuelt@who.int

**Keywords:** adult, immunization, health workers, pregnant women, elderly, chronic diseases

## Abstract

Background/Objective: Historically, immunization programs have focused on infants, children, and women of reproductive age. COVID-19 vaccination prompted countries to vaccinate adults. The objective of this manuscript is to provide a global overview of adult immunization policies post COVID-19 pandemic. Methods: We summarized WHO Strategic Advisory Group of Experts on Immunization (SAGE) recommendations by adult group and analyzed the data reported in 2024 (2023) by WHO Member States (MS) via the WHO/UNICEF electronic Joint Reporting Form on Immunization (eJRF) on national immunization schedules, and from other sources by WHO region and income group. Results: WHO policy recommendations exist for most of the licensed vaccines targeting adults; however, the inclusion in national immunization schedules is higher in high-income (HICs) and middle-income (MICs) countries. For pregnant women, 90% of MS reported vaccination against COVID-19 (65% in low-income countries [LICs]), 63% against tetanus-containing vaccines (73% in LICs), 57% against influenza (4% in LICs), and 21% against pertussis-containing vaccines (all MICs and HICs). For health workers, 91% against COVID-19 (92% in LICs), 59% against influenza (4% in LICs), and 25% against hepatitis B (10% in LICs). For adults with chronic diseases, COVID-19 vaccination data were not available, 58% against influenza, and 23% against pneumococcal disease. For older adults, more than 90% of MS across all income groups reported COVID-19 vaccination, 59% against influenza (8% of LICs versus 89% of HICs), 17% against pneumococcal, and 7% against herpes zoster (HZ). Conclusion: The disparities in adult immunization policies across income groups highlight the need to improve access and strengthen vaccination efforts. A life course approach is essential to maximize the full potential of immunization across all ages.

## 1. Introduction

Historically, national immunization programs, particularly in low- and middle-income countries (LMICs) have focused on infants, children, and women of reproductive age with the aim of preventing disease and mortality in infancy and childhood including tuberculosis, diphtheria, tetanus, pertussis, polio, and measles, among other diseases [1]. With an increasing number of vaccines becoming available for adolescents and adults, the notion of life course immunization is currently being explored by countries around the world.

The COVID-19 pandemic and associated disruptions profoundly impacted global immunization programs, and around 60% of planned mass vaccination campaigns were postponed or canceled in 57 countries as of May 2020 [2]. Global coverage for measles has not yet recovered to pre-pandemic levels [3]. The urgent need to protect other groups against COVID-19—notably older adults, those with chronic conditions, and health workers—prompted many countries to expand their focus beyond childhood immunization and catalyzed significant changes in policies, attitudes, and systems supporting adult vaccination. This has included investments in restructuring governance mechanisms to facilitate collaboration to reach new target groups (e.g., elderly care units, prison health, …), new vaccination sites and identification of synergies with other programs for service delivery, the expansion of the health workforce of vaccinators (e.g., pharmacists, specialists, nurses, …), and the embrace of technological innovations, including the use of new systems allowing for real-time monitoring of vaccination coverage and vaccine safety [4,5]. Some countries with existing seasonal influenza vaccination for adults were able to leverage those programmatic capacities and systems in service of COVID-19 vaccination [6]. By the end of 2023, more than 13.6 billion COVID-19 vaccine doses had been administered globally (mainly to adults), reaching 89% of health workers and 84% of older adults through the end of 2023 [7]. This shows the potential of programs to adapt to life course immunization.

A life course approach to immunization has been defined as a public health approach that states that persons should receive all recommended doses of vaccines through their lives to obtain maximum benefits from vaccine-preventable diseases at different ages, across generations and within their communities [8]. In addition to preventing disease and maximizing health over an individual’s entire lifespan, this approach offers continuous health contact opportunities to promote synergy and integration between immunization operations and other primary health care (PHC) services [9], and contributes to shifting away from a disease-focused approach to a people-centered one [10]. It emphasizes the importance of immunization beyond childhood, addressing the evolving health risks and needs during adolescence, adulthood (including pregnancy), and older age [11]. In the context of aging populations, by 2030, almost 1 billion people will be over 65 years of age [12]; this group is already larger than the number of children younger than five across the world, so it is necessary to invest more in prevention than treatment for healthy aging [13] and to reduce healthcare costs.

In addition, vaccines play a critical role in global health security and pandemic preparedness and have been the cornerstone of the response to six of the seven Public Health Emergencies of International Concern (PHEIC) declared by the WHO to date: H1N1 influenza (2009–2010), polio (2014-ongoing), Ebola (2014–2016 and 2019–2020), COVID-19 (2020–2023) and mpox (2022–2023; 2024-ongoing). Yet, in many countries, particularly in LMICs, gaps in adult immunization platform capacity impeded efforts to achieve the vaccination targets set during the COVID-19 pandemic [14], highlighting the vulnerabilities of existing vaccination systems and reinforcing the need for routine approaches [15] to protect populations across the life course and as preparedness and readiness for future pandemics [16].

This manuscript aims to support the development of a life course approach by providing a detailed global overview of adult immunization. It summarizes current WHO recommendations for vaccines targeting adults and describes the status of adult vaccination policies by population group (pregnant women, health workers, adults with chronic conditions, and older adults), region, and country-income group. These insights contribute to the growing recognition of adult immunization as a critical component of health systems and a cornerstone of the Immunization Agenda 2030 (IA2030) vision: “a world where everyone, everywhere, at every age fully benefits from vaccines for good health and well-being” [17].

## 2. Materials and Methods

### 2.1. Study Design

This study is a review of WHO recommendations for vaccines targeting adults and national adult immunization policies in 194 WHO member states.

### 2.2. Data Sources

We reviewed and summarized existing WHO Strategic Advisory Group of Experts on Immunization (SAGE) [18] recommendations [19] by vaccine and by adult group (pregnant women, health workers, healthy adults not pregnant, adults with chronic conditions, and older adults). We also listed other licensed vaccines for which a WHO SAGE recommendation has not been developed.

This article considers adults to be individuals of 18 years of age and above, recognizing that pregnancy and membership in certain occupational cadres may occur before ‘adulthood’. The definition of ‘health worker’ differs by country; in this manuscript, the term “health workers” refers to all people engaged in work actions whose primary intent is to improve health. This includes health service providers and health management and support workers, working in acute care and long-term care facilities, public health, community-based care, social care and home care, as well as other occupations in the health and social work sectors [20]. While “adults with comorbidities”, “adult with chronic conditions” and “adults with underlying conditions” are all referenced in various adult vaccination recommendations, in this manuscript, we refer to them as “adults with chronic conditions”. Finally, “older adults” is defined by the United Nations as a person who is over 60 years of age [21]; however, the age cut off age varies across countries.

We analyzed data collected via the WHO/UNICEF electronic Joint Reporting Form on Immunization (eJRF) [22] to assess national immunization schedules for vaccines targeting pregnant women, health workers, adults with chronic conditions, and older adults of reporting Member States (MS). We downloaded the data from https://immunizationdata.who.int/ (accessed on 9 January 2025), and analyzed them for all 194 WHO MS and regionally across the six WHO regions: Africa (AFR), 47 MS; Americas (AMR), 35 MS; Eastern Mediterranean (EMR), 21 MS; Europe (EUR), 53 MS; South East Asia (SEAR) 11 MS; and Western Pacific (WPR), 27 MS. The eJRF collects immunization data from countries on an annual basis; in 2024, 191 of 194 (~98%) of MS reported their 2023 data. For those countries for which 2023 data were not available by the time of this analysis, data from the most recent year of reporting were considered (Morocco: 2019, Israel: 2022, and Nauru: 2021).

For COVID-19 vaccination data, we used several data sources for the analysis. We downloaded data from WHO/UNICEF COVID-19 Vaccination Information Hub [23] at https://infohub.crd.co/ (accessed on 4 December 2024). COVID-19 vaccination coverage for health workers and older adults in the period 2021–2023 was used as proxy of COVID-19 vaccination policies targeting those groups. Since this source included limited information of vaccination policy of pregnant women against COVID-19, we used data from COVID-19 Maternal Immunization Tracker (COMIT) [24] (https://www.comitglobal.org/), accessed on 10 December 2024) from Berman Institute of Bioethics & Center for Immunization Research, Johns Hopkins University. This source summarizes policies on COVID-19 vaccination for pregnant and lactating women. It was last updated on 05 January 2023. For this analysis, countries with policies recommending or permitting COVID-19 vaccination for pregnant women issued by public health authorities or regulatory agencies were considered. The latest country-reported data from WHO/UNICEF COVID-19 vaccination information hub for Q1–Q2 2024 [23], regional COVID-19 vaccination dashboards [25,26], and official reports from WHO regional offices [27,28] were used to supplement and triangulate the COVID-19 vaccination results for health workers, older adults, and pregnant women. Eritrea and Democratic People’s Republic of Korea were considered as not vaccinating against COVID-19 based on reports from WHO regional and country offices. Overall, data on COVID-19 vaccination were reported by 168 MS for health workers, 177 for older adults, and 186 for pregnant women at least once from January 2021 to June 2024. Data on COVID-19 vaccination policies of adults with chronic conditions were not included in the analysis due to insufficient country reporting.

For RSV, both maternal vaccines and vaccines for older adults were first licensed in 2023 and only started to be used by countries at the end that year; consequently, this was not captured in 2023 eJRF reporting. Therefore, we relied on information provided by vaccine manufacturers and available in the public domain on which countries had started to use these products.

For this analysis, we additionally used the latest World Bank income group classifications (HIC: high-income economies, UMIC: upper-middle income economies, LMIC: lower-middle-income economies, and LIC: low-income economies) as listed in November 2024 [29].

### 2.3. Bias

The completeness and accuracy of data from eJRF rely on the quality of reporting by countries. Additional efforts to complete missing data were conducted to reduce the risk of information bias.

### 2.4. Statistical Analysis

Analyses were conducted using R Studio software version 2023.03.0+386, and maps for data visualizations were developed using QGIS software version 3.40 according to WHO Operating Procedures for maps.

## 3. Results

### 3.1. Vaccines Targeting Adults

Table 1 presents a summary of recommendations for vaccines targeting adults in two categories: (1) for all immunization programs and (2) for programs with certain characteristics (i.e., funding and programmatic capacity). Table 2 presents a summary of the vaccine-preventable diseases against which an adult is recommended to be protected through vaccination (if not previously immunized) as well as vaccines recommended for some high-risk adult populations or adults in/travelling to certain regions. Where available, WHO SAGE recommendations for the different adult groups are described and referenced. 

### 3.2. Status of Immunization Policies for Pregnant Women

The WHO provides guidance for the vaccination of pregnant women where there is specific interest to protect the mother or the newborn. As reflected in Table 1, vaccines against COVID-19, seasonal influenza, pertussis, and RSV are recommended for pregnant women. Tetanus vaccination is also recommended if the mother did not receive either six TTCV doses during childhood or five doses if first vaccinated during adolescence/adulthood before the time of reproductive age. Other vaccines can be also recommended to pregnant women at high risk of exposure as summarized in Table 2.

**Figure 1:**
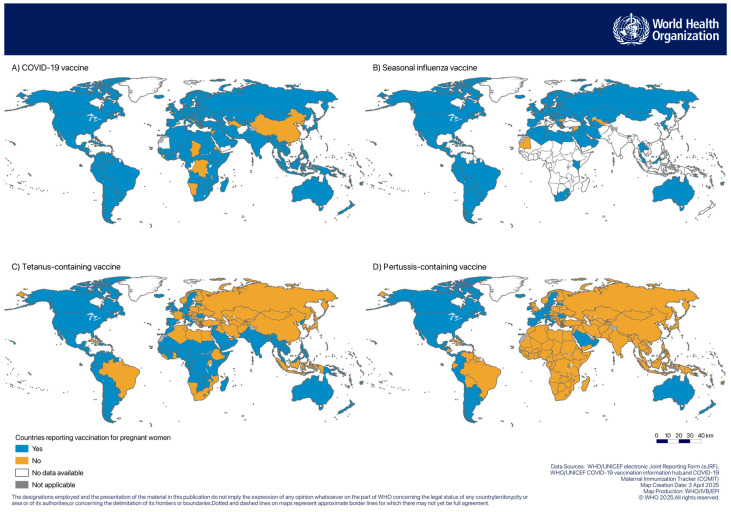
Geographical distribution of countries that reported vaccination for pregnant women with (**A**) COVID-19 vaccine, (**B**) seasonal influenza vaccine, (**C**) tetanus-containing vaccine, and (**D**) pertussis-containing vaccine.

Based on 2023 data, 90% of 194 WHO MS were identified as recommending vaccinating pregnant women against COVID-19, 57% against seasonal influenza, 63% with tetanus-containing vaccine, and 21% with pertussis-containing vaccine (Table 3). Figure 1 shows the geographical distribution of countries reporting vaccination with these four vaccines.

As mentioned above, countries did not report RSV in the 2023 eJRF. However, as of end of 2024 some countries had started to use RSV maternal immunization in their national programs, including seven countries in Europe and three in the Americas, Communication from Pfizer, January 2025.

### 3.3. Status of Immunization Policies for Health Workers

The WHO recommends an annual revaccination of health workers against two antigens: COVID-19 and seasonal influenza. Also, following WHO guidance [57], health workers should be vaccinated against nine additional antigens if not previously immunized: Bacillus Calmette–Guérin (BCG), diphtheria, hepatitis B, measles, meningococcal, pertussis, polio, rubella, and varicella. In addition, in appropriate high-incidence settings (including emergencies), other vaccines should also be considered for health workers as described in Table 2.

Based on 2023 data, 91% of 194 MS reported vaccinating health workers against COVID-19, 59% against seasonal influenza, 24% against hepatitis B, 6% against measles-rubella, and 5% against varicella (Table 4). Other vaccines that were reported by fewer number of countries included those against polio, meningococcal disease, mpox, pneumococcal disease, hepatitis A, BCG, tetanus, diphtheria and pertussis.

### 3.4. Status of Immunization Policies for Adults with Chronic Conditions

The WHO recommends annual revaccination against COVID-19 and seasonal influenza for adults with chronic conditions. Additional vaccines, such as those against pneumococcal disease and hepatitis B, may be advised based on individual health risks, as described in Table 2.

Limited information is reported in the eJRF regarding vaccination of people with chronic conditions. The two main vaccines reported by countries for this group were seasonal influenza and pneumococcal vaccine (Table 5). Overall, 113 countries (58% of MS) report vaccination against seasonal influenza for high-risk groups. Among them, 105 countries report “vaccination for adults with chronic conditions”, 94 report vaccination for “people living in long-term care facilities”, and 69 report vaccination for “other groups”.

Concerning vaccination against pneumococcal disease, 45 countries (23% of MS) reported vaccination for high-risk groups. Among them, 32 countries reported “high-risk groups” (generic term), eight reported “chronic conditions” (generic term), 10 reported specific conditions (for example, asplenia), 5 reported “immunocompromised individuals” (generic term), and 3 target “people living in long-term care facilities”.

### 3.5. Status of Immunization Policies for Older Adults

The WHO recommends annual revaccination against COVID-19 and seasonal influenza for older adults. Pneumococcal vaccination for older adults can also be considered. Additional vaccines, such as HZ (expected update of WHO recommendation in 2025) and RSV, are also available (Table 1).

**Figure 2:**
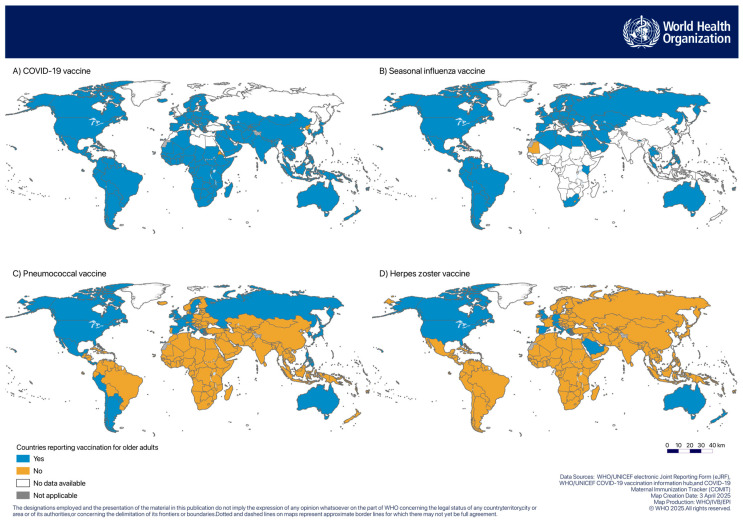
Geographical distribution of countries that reported vaccination for older adults with (**A**) COVID-19 vaccine, (**B**) seasonal influenza vaccine, (**C**) pneumococcal vaccine, and (**D**) HZ vaccine.

Based on 2023 data, 94% of 194 WHO MS reported vaccinating against COVID-19, 59% against seasonal influenza, 17% against pneumococcal disease (mainly in the Americas and Europe), and 7% against HZ (Table 5). Figure 2 shows the geographical distribution of countries reporting vaccination with these four vaccines.

In 2024, no information about other recently licensed vaccines was reported in eJRF by countries. However, by the end of 2024, five European countries, one from the Western Pacific, and two from the Americas (all HICs) had introduced the RSV vaccine for older adults. (Communication from Pfizer and GSK, January 2025).

## 4. Discussion

Adult immunization policy recommendations exist for most available vaccines; however, inclusion in national immunization schedules has predominantly been reported in HICs and MICs. The global COVID-19 vaccination effort, which saw 99% of countries introduce the vaccine, brought adult immunization to the forefront of national immunization programmes. The COVID-19 experience can have lasting effects on how countries, particularly LMICs, approach immunization programs, encouraging a shift from the traditional childhood-centric focus to a life course approach to immunization [58].

The maternal immunization approach had been used for many years across geographies and income groups prior to the COVID-19 pandemic across a wide range of countries. This is a main component of the WHO’s maternal and neonatal tetanus elimination (MNTE) strategy [59]. To date, 80% of LMICs and 73% of LICs are offering tetanus-containing vaccines to pregnant women. During the pandemic, 90% of countries (65% of LICs) targeted pregnant women with vaccines against COVID-19. Looking to other antigens, 57% of the countries (only 4% of LICs) report vaccinating against seasonal influenza, and only 21% (mainly HICs) are using tetanus-pertussis-containing vaccines to protect infants against emerging pertussis cases. Looking forward, a number of vaccines targeting pregnant women are expected to be available and accessible in the coming years for other pathogens causing high burden, notably RSV (claims the lives of more than 45,000 young infants worldwide each year) [60], and group B streptococcus (GBS), the leading cause of neonatal/infant sepsis and meningitis globally (around 91,000 infant deaths and 46,000 stillbirths per year) [61]. Gavi, the Vaccine Alliance (Gavi) has already committed to an in-principle investment for maternal RSV and GBS vaccine introduction in Gavi-eligible countries in the near future [62]. These new and existing vaccines further represent an opportunity to strengthen maternal immunization as a platform for improved quality of care during antenatal care visits.

Despite 91% of countries reporting vaccinating health workers against COVID-19, fewer countries report having policies for this group for other antigens. Fifty-nine percent of countries report vaccinating health workers against seasonal influenza, though only 4% of LICs reported as such, compared with 89% of HICs. More than 85% of countries in the European and Americas regions include seasonal influenza vaccination for health workers in their national schedules. Vaccination of health workers against other antigens such as hepatitis B, measles and rubella or varicella is mainly reported by HICs. Vaccines targeting health workers play a critical role in safeguarding the health workforce while also reducing the likelihood of transmission to patients. In addition, health workers play a key role in recommending vaccines to other key groups. Given this, targeted strategies to effectively engage, protect, and empower this group on communicating the benefits of adult immunization must be prioritized [63].

Similarly, while more than 90% of MS across all income groups vaccinated older adults against COVID-19, the situation for other vaccines targeting this population groups is heterogeneous, with important differences observed between regions and income groups. For seasonal influenza, for instance, 59% of reporting MS indicate vaccinating this group (8% of LICs and 30% of LMICs compared with 89% of HICs). Pneumococcal and HZ vaccination for older adults is reported to be part of national immunization schedules by even fewer countries, 17% and 7%, respectively, with inclusion limited to the Americas, Eastern Mediterranean, European and Western Pacific regions. While each of these vaccines has the potential to reduce the burden of preventable diseases among older adults, their inclusion in national programs remains inconsistent. As older adults constitute increasingly large proportions of national populations, vaccination efforts targeting this group will be a key strategy for improving long-term health while reducing burden on health systems [64]. To unlock the full potential of vaccines for this group, further study is required to understand the unique immunological, socio-behavioral, programmatic adaptations, and full economic value that influence vaccination efforts in these groups [63].

In 2024, the WHO, UNICEF, Gavi, and the Gates Foundation launched a global advocacy campaign alongside World Immunization Week: “Humanly Possible”. This campaign aims to celebrate the 50th anniversary [65] of the Expanded Programme on Immunization (EPI) and inform the vision for the future of immunization. In the coming years, it is expected that a number of new vaccines targeting groups beyond childhood will become available, such as next generation of tuberculosis vaccines, group B streptococcus vaccines, cytomegalovirus vaccine and combination vaccines for respiratory viruses, among others. With the emergence of new platform technologies, such as mRNA, vaccine candidates can advance into clinical testing at a much faster rate, shortening development timelines. Mechanisms to facilitate access to existing and new products in LMICs will be required as well as t, strong adult immunization platforms. [66]. New innovations in vaccine delivery such as heat-stable micro-array patches and digital health tools like mobile phone reminders could facilitate vaccine uptake. However, these new vaccines and delivery innovations will need to be affordable and cost-effective to countries. An analysis of lessons learned from countries with successful adult vaccination programs could identify which innovations have proven to be the most successful and cost-effective, but would need to be applicable to LMIC contexts. National Immunization Technical Advisory Groups (NITAGs) or equivalent bodies will need to broaden their expertise to assess new products for potential inclusion in immunization schedules across all age groups, considering national priorities and the capacity of health systems. At the same time, governments will need to prioritize, where relevant, vaccines targeting adults as part of their National Immunization Strategy processes, while ensuring alignment with broader public health goals.

In preparation for these anticipated advancements in life course vaccines, we need to ensure there is a solid foundation upon which to build. A proactive approach to designing immunization schedules and investing in programs that include adults more comprehensively is needed, considering specific adult metrics to estimate public health impact, economic impact (at both the individual- and society-levels), and impact on health systems. While data from high and upper middle-income countries highlight the socio-economic value of adult immunization programs based on the use of four vaccines (seasonal influenza, respiratory syncytial virus (RSV), herpes zoster and pneumococcal diseases), which can return up to 19 times their initial investment to society [67], additional data, especially from LMICs, is crucial to fully assess the potential and impact of these programs.

It will be also important to establish tailored adult delivery strategies as part of the PHC services (e.g., antenatal care, adult/older adult care) and leveraging EPI structure through which vaccines are offered regularly by knowledgeable health workers and where the adult population can seek to be vaccinated. Additionally, in the current environment, and considering the stagnation of global childhood immunization coverage, addressing misinformation—particularly in the wake of the COVID-19 pandemic and the rise of anti-vaccine movements—will be crucial to sustaining public trust in vaccines and generating demand among adult populations. Engaging community leaders and healthcare professional networks will be essential for developing tailored communication for adult populations. Finally, these strategies will need to be accompanied by indicators and electronic systems (such as leveraging COVID-19 vaccine innovations), adapted to track adult immunization progress as part of the life course immunization approach and that are interoperable with other registries. Without adequate coverage, the effectiveness and impact of these programs will be compromised.

Our analysis is based on country reports to WHO and other sources and has some limitations. While the reporting in the national immunization schedules section of the eJRF follows a standardized format for all target groups, the use of open text fields for vaccines recommended for high-risk groups introduces ambiguity. This format, intended to reduce the reporting burden on MS, may lead to overlapping information between children and adult groups, making it difficult to analyze data by specific subcategories of risk groups. Furthermore, while the data reported in the schedule for all vaccines, except for seasonal influenza, are prepopulated each reporting year—requiring countries to update or add new information annually—the data on seasonal influenza target groups are not prepopulated. As a result, countries must enter this information annually, which may introduce inconsistencies or gaps in the data. While nearly all MS reported data for 2023 through the eJRF (191 of a total of 194 WHO MS), COVID-19 vaccination data were not yet being collected through this system, and the number of countries that reported COVID-19-related data during the 2021 to 2023 was fewer. At the same time, the numbers of countries currently vaccinating adult groups against COVID-19 might be already lower than the 2023 reported data. In addition, given the evolving epidemiology and the need for adapted vaccines targeting current circulating variants [68], the sunset of Gavi COVID-19 program at the end of 2025 [69], and the decreased demand of COVID-19 vaccines, the mid-to-long term sustainability of COVID-19 vaccination particularly in LMICs is uncertain. Another limitation is that the analysis primarily relies on stratification by income group, as the reported data do not allow for assessing a broad range of factors such as country-specific policy priorities, cultural norms, or infrastructure limitations, which likely influence decisions to adopt vaccine policies for adult populations.

## 5. Conclusions

The use of existing adult-targeted vaccines, coupled with the COVID-19 vaccination experience, has created an opportunity to advance adult immunization. With more vaccines expected for adults in the coming decade, and as evidence to understand their full value continues to grow, the concept of life course immunization is set to become a social norm. Improving access and integrating adult vaccination into national immunization strategies and primary health care promises to unlock the full potential of immunization across all ages and regions.

## Figures and Tables

**Table 1:** Summary of recommendations for vaccines targeting adults.

Antigen	Pregnant Women	Health Workers (HWs) *	Healthy Adults (Not Pregnant)	Adults with Chronic Conditions	Older Adults	Considerations	Source Description
**For all immunization programmes**							
COVID-19 [30]	x	x	x	x	x	One-dose primary series for those that have never received a COVID-19 vaccine and revaccination 6–12 months later for high priority-use groups and sub-populations with special considerations for COVID-19 vaccination (older adults, adults with comorbidities or severe obesity, adults with immunocompromising conditions, and health workers). For pregnant women, a single dose is recommended in each pregnancy regardless of previous vaccination status; ideally during the second trimester or at any opportunity	WHO SAGE Roadmap for prioritizing uses of COVID-19 vaccines, 2023 Link: https://iris.who.int/handle/10665/373987 (accessed on 10 October 2024)
**For programmes with certain characteristics (i.e., funding and programmatic capacity)**							
Seasonal influenza [31]	x	x		x	x	One dose, annual revaccination WHO recommended target groups for “countries considering the initiation or expansion of seasonal influenza vaccination”. “Depending on national disease goals, capacity and resources, epidemiology, national policies and priorities, and disease burden, countries may consider additional (sub)populations for vaccination”.	Vaccines against Influenza: WHO Position Paper—May 2022. Link: https://iris.who.int/handle/10665/354265 (accessed on 10 October 2024)
Herpes zoster (HZ) [32]					x	Two doses WHO has not provided a recommendation concerning the routine use of HZ vaccine yet (expected reco—mmendation in 2025). “However, countries with an aging population and demographic shift towards older ages, may decide to introduce routine HZ vaccination if they have an important burden of disease and consider the programme beneficial”.	Varicella and Herpes Zoster Vaccines: WHO Position Paper, June 2014. Link: https://iris.who.int/handle/10665/242227 (accessed on 10 October 2024)
Pertussis [33]	x	x	x			“1 dose of Tdap (in the 2nd or 3rd trimester and preferably at least 15 days before the end of pregnancy) as a strategy additional to routine primary infant pertussis vaccination in countries or settings with high or increasing infant morbidity/ mortality from pertussis”. “High coverage in infants through routine immunization should be in place prior to the introduction of vaccination in adolescents and adults”. “[HWs] should be prioritized as a group to receive pertussis vaccine”.	Pertussis Vaccines: WHO Position Paper—August 2015. Link: https://iris.who.int/handle/10665/242413 (accessed on 10 October 2024)
Pneumococcal [34]				x	x	“In countries that have a mature childhood pneumococcal immunization programme, decisions about initiating such a programme in adults should take into account local disease burden and cost-effectiveness considerations”.	Considerations for Pneumococcal Vaccination in Older Adults, 2021 Link: https://iris.who.int/handle/10665/341722 (accessed on 10 October 2024)
Respiratory Syncytial Virus (RSV) [35]	x				x **	One dose “A single vaccine dose in the third trimester of pregnancy, as defined in the local context (≥28 weeks of gestation in most settings)”.	Meeting of the Strategic Advisory Group of Experts on Immunization, September 2024 Link: https://iris.who.int/handle/10665/379718 (accessed on 10 October 2024)

Note: Antigens are presented in alphabetical order. * All health workers should be up-to-date with immunization as recommended in their national immunization schedule. ** Despite the availability of RSV vaccine for older adults, there is not yet a WHO SAGE recommendation for use in this group.

**Table 2:** Summary of vaccine-preventable diseases against which an adult is recommended to be protected through vaccination (if not previously immunized) and vaccines recommended for some high-risk adult populations or adults in/travelling to certain regions.

Antigen	Pregnant Women	Health Workers (HWs) *	Healthy Adults (Not Pregnant)	Adults with Chronic Conditions	Older Adults	Considerations	Source Description
**For adults to be protected (if not previously immunized, natural or vaccination)**							
BCG [36]		x				One dose For “unvaccinated, TST [Tuberculin Skin Test] or IGRA [Interferon gamma release assay test] -negative… adults from settings with high incidence of TB and/or high leprosy burden”. For “unvaccinated TST- or IGRA-negative… adults moving from low to high TB incidence/ leprosy burden settings”. For “unvaccinated TST- or IGRA-negative persons at risk of occupational exposure in low and high TB incidence areas (e.g., health-care workers, laboratory workers, medical students, prison workers, other individuals with occupational exposure)”, travelers, and returning migrants.	BCG Vaccines: WHO Position Paper—February 2018. Link: https://iris.who.int/handle/10665/260307 (accessed on 18 December 2024)
Diphtheria [37]		x	x	x		Three doses primary series and two subsequent booster doses.	Diphtheria Vaccines: WHO Position Paper—August 2017. Link: https://iris.who.int/handle/10665/258683 (accessed on 18 December 2024)
Hepatitis B [38]		x	x	x **		Three doses “Catch up of unvaccinated cohorts (if the necessary resources are available)”.	Hepatitis B Vaccines: WHO Position Paper—July 2017. Link: https://iris.who.int/handle/10665/255873 (accessed on 18 December 2024)
Measles [39]		x	x			Two doses “Vaccine should be offered to… adults likely or known to be susceptible [including travelers]”.	Measles Vaccines: WHO Position Paper—April 2017. Link: https://iris.who.int/handle/10665/255705 (accessed on 18 December 2024)
Polio [40]		x	x			“Before travelling abroad, persons residing in countries with active transmission of a wild or vaccine-derived poliovirus should complete a full course of polio vaccination in compliance with their national schedule and receive 1 dose of IPV or bOPV within 4 weeks to 12 months of travel”.	Polio Vaccines: WHO Position Paper—June 2022. Link: https://iris.who.int/handle/10665/357168 (accessed on 18 December 2024)
Rubella [41]		x	x			One dose Women of reproductive age but contraindicated during pregnancy.	Rubella Vaccines: WHO Position Paper—July 2020. Link: https://iris.who.int/handle/10665/332952 (accessed on 18 December 2024)
Tetanus [42]	x		x			“Vaccination history should be verified in order to determine whether a dose of TTCV [Tetanus Toxoid Containing Vaccine] is needed in the current pregnancy”. “Pregnant women and their newborn infants are protected from birth-associated tetanus if the mother received either 6 TTCV doses during childhood or 5 doses if first vaccinated during adolescence/adulthood (documented by card, immunization registry and/or history) before the time of reproductive age”.	Tetanus Vaccines: WHO Position Paper—February 2017. Link: https://iris.who.int/handle/10665/254583 (accessed on 10 October 2024)
Varicella [32]		x	x	x		Two doses “Varicella vaccine is usually contraindicated in persons with congenital or acquired immune deficiencies. However, due to the increased severity of varicella in certain groups of immunocompromised persons, varicella vaccination may be considered in these groups. Combination MMRV [Measles-Mumps-Rubella-Varicella] vaccine has not been tested and is contraindicated in immunocompromised persons”. Contraindicated during pregnancy.	Varicella and Herpes Zoster Vaccines: WHO Position Paper, June 2014. Link: https://iris.who.int/handle/10665/242227 (accessed on 10 October 2024)
**For some high-risk adult populations or adults in/travelling to certain regions**							
Cholera [43]	x	x	x	x		Two doses In areas at risk or where endemic cholera is present. Pregnant and lactating women and HIV-infected individuals should be included in Oral Cholera Vaccine (OCV) campaigns. OCV should be considered for travelers at high risk of infection. “OCV should be considered for emergency and relief workers who are likely to be directly exposed to cholera patients or to contaminated food or water, particularly those staying in areas with poor access to health-care facilities. Other health-care workers are generally not at special risk of cholera”.	Cholera Vaccines: WHO Position Paper—August 2017. Link: https://iris.who.int/handle/10665/258764 (accessed on 18 December 2024)
Dengue [44]			x	x		Two doses “Persons with comorbidities, [such as sickle cell anaemia, diabetes, hypertension, or underlying comorbidities that may result in bleeding tendencies (e.g., ulcerative colitis)] who live in dengue-endemic countries could be offered vaccination. Until more data on efficacy-safety profiles become available, WHO recommends the upper limit of 60 years for vaccination”. “Persons living in non-endemic countries who have previously been infected with any of the 4 dengue virus serotypes following travel to dengue-endemic countries, may benefit from vaccination to prevent a second (and hence potentially more severe) dengue infection when travelling again to an endemic country”.	WHO Position Paper on Dengue Vaccines—May 2024. Link: https://iris.who.int/handle/10665/376642 (accessed on 18 December 2024)
Ebola [45]	x	x	x	x		One dose Countries with Ebola outbreaks: ring vaccination targeting contacts and contacts of contacts, including pregnant women. Countries at risk of Ebola outbreaks: preventive vaccination of health workers.	Extraordinary meeting of the Strategic Advisory Group of Experts on Immunization on Ebola vaccination, May 2024: conclusions and recommendations. Link: https://iris.who.int/handle/10665/378109 (accessed on 18 December 2024)
Hepatitis A [46]			x ***	x ***		Two doses if >40 years of age “Groups at higher risk of hepatitis A should be vaccinated”.	WHO position paper on hepatitis A vaccines—October 2022 Link: https://iris.who.int/handle/10665/363397 (accessed on 18 December 2024)
Hepatitis E [47]	x	x	x			One dose “The use of the vaccine to mitigate or prevent outbreaks should be considered as well to mitigate consequences in high-risk groups such as pregnant women”.	Hepatitis E vaccine: WHO position paper, May 2015 Link: https://iris.who.int/handle/10665/242352 (accessed on 18 December 2024)
Japanese encephalitis (JE) [48]	x		x			“If JE risk is sufficient to warrant vaccination of pregnant women, inactivated vaccines should be used preferentially”. “For travelers to endemic areas with extensive outdoor exposure during the transmission season”.	Japanese Encephalitis Vaccines: WHO Position Paper, 2015. Link: https://iris.who.int/handle/10665/242325 (accessed on 18 December 2024)
Meningococcal [49,50,51]		x				One booster dose 3–5 years after the primary dose may be given to persons considered to be at continued risk of exposure.	Meningococcal Vaccines: WHO Position Paper—November 2011. Link: https://iris.who.int/handle/10665/241846 (accessed on 10 October 2024) Meningococcal A Conjugate Vaccine: Updated Guidance, February 2015. Link: https://iris.who.int/handle/10665/242320 (accessed on 10 October 2024) Meningococcal Vaccines: WHO Position Paper on the Use of Multivalent Meningococcal Conjugate Vaccines in Countries of the African Meningitis Belt, January 2024. Link: https://iris.who.int/handle/10665/375624 (accessed on 18 December 2024)
Mpox [52]	x	x	x ****	x	x	One or two doses (depending on the vaccine product) “Primary preventive vaccination is recommended for laboratory personnel working with orthopoxviruses and periodic revaccination should be considered”. “For persons at high risk of exposure to mpox during an outbreak”.**** During pregnancy, where consideration is given to vaccination, non-replicating vaccine … should be used.	Smallpox and Mpox (Orthopoxviruses): WHO Position Paper, August 2024. Link: https://iris.who.int/handle/10665/378526 (accessed on 10 October 2024)
Rabies pre-exposure prophylaxis (PrEP) [53]			x			Two doses “Sub-populations in highly endemic settings with limited access to timely and adequate PEP [Post Exposure Prophylaxis], individuals at occupational risk, and travelers who may be at risk of exposure”. “PrEP may be considered for medical professionals who regularly provide care to persons with rabies”.	Rabies Vaccines: WHO Position Paper—April 2018. Link: https://iris.who.int/handle/10665/272372 (accessed on 18 December 2024)
Tick-borne encephalitis [54]	x		x		x	Three doses followed by additional booster doses for those who will continue to be at risk “The vaccine should be used in pregnant women who live in areas where the incidence of the disease is high (>5 cases/100,000 population per year)”. “People travelling from nonendemic areas to endemic areas should be offered vaccination if their visits will include extensive outdoor activities”.	Vaccines against Tick-Borne Encephalitis: WHO Position Paper—2011. Link: https://iris.who.int/handle/10665/241769 (accessed on 18 December 2024)
Typhoid [55]			x			One or more doses depending on the product “Catch-up vaccination with TCV [Typhoid Conjugate Vaccine] up to 15 years of age is recommended when feasible and supported by epidemiologic data”. “Travelers from non-endemic to endemic areas”. “In typhoid-endemic areas, professional food handlers can be vaccinated against typhoid”. “Clinical microbiology laboratory staff with a recognized risk of occupational exposure to S. Typhi should be offered vaccination against typhoid”.	Typhoid Vaccines: WHO Position Paper—March 2018. Link: https://iris.who.int/handle/10665/272273 (accessed on 18 December 2024)
Yellow fever [56]			x			One dose Individuals in endemic countries and travelers to these countries. There is currently no recommendation regarding HWs.	Vaccines and Vaccination against Yellow Fever: WHO Position Paper—June 2013. Link: https://iris.who.int/handle/10665/242089 (accessed on 18 December 2024)

Note: Antigens are presented in alphabetical order. * All health workers should be up-to-date with immunization as recommended in their national immunization schedule. ** Hepatitis B: Adults with chronic definition includes “patients who frequently require blood or blood products, dialysis patients, diabetes patients, recipients of solid organ transplantation, person with chronic liver disease including those with Hepatitis C, person with HIV infection, men who have sex with men, persons with multiple sexual partners, as well as health workers and others who may be exposed to blood, blood products or other potentially infectious body fluids during their work”. *** Hepatitis A: High risk groups include “[travelers] from low-endemic countries to areas of intermediate or high endemicity, men who have sex with men, at-risk occupational groups (such as sewage workers or laboratory personnel handling hepatitis A virus specimens), people who inject drugs, people who experience homelessness, migrants, refugees, incarcerated persons; and patients with chronic liver disease or people living with HIV, particularly in countries with low and very low endemicity”. **** Mpox: in the context of an mpox outbreak, vaccination is recommended for persons at high risk of exposure. Populations to consider for vaccination may include: “based on local epidemiology, members of a defined area or community (e.g., village), with a documented high risk of exposure to persons with mpox; sex workers; gay, bisexual or other men who have sex with men (MSM) with multiple sexual partners; or other individuals with multiple casual sexual partners; health workers at risk of repeated exposure; clinical laboratory and health-care personnel performing diagnostic testing for mpox or providing care; and outbreak response team members (as designated by national public health authorities); contacts of persons with mpox, ideally within 4 days of first exposure”.

**Table 3:** Status of immunization policies for pregnant women by WHO region and income group.

		Number of Member States (MS)	COVID-19 Vaccine (%)	Seasonal Influenza Vaccine (%)	Tetanus-Containing Vaccine ** (%)	Pertussis-Containing Vaccine (Tdap/aP) (%)
Number of reporting countries		194	186 (96%)	116 (60%)	194 (100%)	194 (100%)
WHO Region	Africa	47	35 (74%)	4 (9%)	37 (79%)	1 (2%)
	Americas	35	35 (100%)	31 (89%)	30 (86%)	16 (46%)
	Eastern Mediterranean	21	17 (81%)	14 (67%)	12 (57%)	2 (10%)
	Europe	53	52 (98%)	48 (91%)	17 (32%)	17 (32%)
	South East Asia	11	10 (91%)	4 (36%)	9 (82%)	0
	Western Pacific	27	25 (93%)	9 (33%)	17 (63%)	4 (15%)
Country-income group	HIC	63	62 (100%)	54 (87%)	37 (60%)	28 (45%)
	UMIC	53	49 (92%)	42 (79%)	24 (45%)	10 (19%)
	LMIC	50	43 (86%)	12 (24%)	40 (80%)	1 (2%)
	LIC	25	17 (65%)	1 (4%)	19 (73%)	0
	Not classified *	3	3	1	2	1
All regions		194	174 (90%)	110 (57%)	122 (63%)	40 (21%)

* Not included in the WB income classification: Niue and Cook Islands. Not classified by WB due to unavailability of data: Venezuela. ** Includes Td, TT, Td-IPV, Tdap, and Tdap-IPV.

**Table 4:** Status of immunization policies for health workers by WHO region and country income- group.

		Number of Member States (MS)	COVID-19 Vaccine (%)	Seasonal Influenza Vaccine (%)	Hepatitis B Vaccine (%)	Measles-Rubella Containing Vaccines (%)	Varicella Vaccine (%)
Number of reporting countries		194	178 (92%)	119 (61%)	194 (100%)	194 (100%)	194 (100%)
WHO Region	Africa	47	46 (98%)	5 (11%)	2 (4%)	0 (0%)	0 (0%)
	Americas	35	35 (100%)	32 (91%)	21 (60%)	3 (9%)	4 (11%)
	Eastern Mediterranean	21	13 (62%)	14 (67%)	5 (24%)	1 (5%)	2 (10%)
	Europe	53	45 (85%)	51 (96%)	17 (32%)	5 (9%)	3 (6%)
	South East Asia	11	10 (91%)	4 (36%)	1 (9%)	1 (9%)	0 (0%)
	Western Pacific	27	27 (100%)	9 (33%)	2 (7%)	1 (4%)	0 (0%)
Country-income group	HIC	63	52 (84%)	55 (89%)	23 (37%)	5 (8%)	6 (10%)
	UMIC	53	51 (96%)	42 (79%)	19 (36%)	4 (8%)	3 (6%)
	LMIC	50	46 (92%)	15 (30%)	5 (10%)	1 (2%)	0 (0%)
	LIC	25	24 (92%)	1 (4%)	0 (0%)	0 (0%)	0 (0%)
	Not classified *	3	3	2	1	1	0
	All regions	194	176 (91%)	115 (59%)	48 (25%)	11 (6%)	9 (5%)

* Not included in the WB income classification: Niue and Cook Islands. Not classified by WB due to unavailability of data: Venezuela.

**Table 5:** Status of immunization policies for adults with chronic conditions and older adults by WHO Region and country income group.

		Number of Member States (MS)	COVID-19 Vaccine (%)	Seasonal Influenza Vaccine (%)		Pneumococcal Vaccine ** (%)		HZ Vaccine (%)
			Older Adults	Older Adults	Adults with Chronic Conditions	Older Adults	Adults with Chronic Conditions	Older Adults
Number of reporting countries		194	184 (95%)	118 (61%)	116 (60%)	194 (100%)	194 (100%)	194 (100%)
WHO Region	Africa	47	46 (98%)	5 (11%)	4 (9%)	0	0	0
	Americas	35	35 (100%)	32 (91%)	32 (91%)	12 (34%)	13 (37%)	2 (6%)
	Eastern Mediterranean	21	15 (71%)	15 (71%)	15 (71%)	1 (5%)	7 (33%)	2 (10%)
	Europe	53	50 (94%)	49 (92%)	50 (94%)	16 (30%)	22 (42%)	7 (13%)
	South East Asia	11	9 (82%)	4 (36%)	4 (36%)	0	0	0
	Western Pacific	27	27 (100%)	9 (33%)	8 (30%)	4 (15%)	3 (11%)	2 (7%)
Country-income group	HIC	63	58 (94%)	55 (89%)	55 (89%)	25 (40%)	32 (52%)	13 (21%)
	UMIC	53	51 (96%)	40 (75%)	41 (77%)	7 (13%)	12 (23%)	0
	LMIC	50	46 (92%)	15 (30%)	13 (26%)	1 (2%)	1 (2%)	0
	LIC	25	24 (92%)	2 (8%)	2 (8%)	0	0	0
	Not classified *	3	3	2	2	0	0	0
	All regions	194	182 (94%)	114 (59%)	113 (58%)	33 (17%)	45 (23%)	13 (7%)

Notes: * Not included in the WB classification: Niue and Cook Islands. Not classified by WB due to unavailability of data: Venezuela. ** Includes those countries reporting use of PCV13, PCV15, PCV20, and/or PPV23.

## Data Availability

The original data presented in the study are openly available in WHO Immunization Data portal at https://immunizationdata.who.int/ (accessed on 9 January 2025), WHO/UNICEF COVID-19 vaccination information hub at https://infohub.crd.co/ (accessed on 4 December 2024), and COVID-19 Maternal Immunization Tracker (COMIT) from the Berman Institute of Bioethics & Center for Immunization Research, Johns Hopkins University at https://www.comitglobal.org/about/data (accessed on 10 December 2024).
